# Multilevel factors predicting medication adherence among gout patients in China: A 1‑year prospective cohort study

**DOI:** 10.1097/MD.0000000000050676

**Published:** 2026-09-18

**Authors:** Haiyan Jiang, Yanda Yang, Xuan Lei, Ting Yi, Xiran Luo, Weiyu Yang, Shuya Xiong, Rong Wang, Yuanjun Xie, Tao Lu, Liwen Yao, Zhaoxia Ran, Zepeng Lai, Yapeng Lin

**Affiliations:** a Department of Rheumatology and Immunology, The First Affiliated Hospital of Chengdu Medical College, Chengdu, Sichuan, China; b School of Clinical Medicine, Shanghai University of Medicine & Health Sciences, Shanghai, China; c School of Clinical Medicine, Chengdu Medical College, Chengdu, Sichuan, China; d Department of Medical Affairs, The People’s Hospital of Jianyang City, Jianyang, Sichuan, China.

**Keywords:** adherence, China, gout, predictors, prospective cohort study

## Abstract

Adherence to urate-lowering therapy (ULT) is crucial for gout management, yet it remains suboptimal worldwide, particularly in China where relevant data are scarce. This study investigates the prevalence and predictors of medication adherence among Chinese gout patients receiving ULT over a 1-year period. A 1-year prospective cohort study was conducted among gout patients prescribed ULT at a single center. Adherence was assessed using the Compliance Questionnaire in Rheumatology at 6 and 12 months, with scores ≥80% defining adherence. Predictors encompassing socioeconomic, therapy-related, patient-related, condition-related, and healthcare system–related factors were analyzed using multiple linear regression models. Among 141 recruited patients (96.5% male), the adherence prevalence was 8.3% (11/133) at 6 months and 7.1% (9/127) at 1 year. In multiple linear regression, a higher gout knowledge (Gout Knowledge Questionnaire) score was independently associated with better adherence at 6 months (β = −0.223, *P* = .001). At 1 year, taking urate-lowering agent in the past week was the sole independent predictor (β = 0.178, *P* = .014). Medication adherence to ULT among gout patients in this Chinese cohort is notably low and declines over time. Patient knowledge and recent medication-taking behavior are key modifiable predictors. Interventions should be targeted within the first 6 months of therapy.

## 1. Introduction

Gout, one of the most prevalent forms of inflammatory arthritis,^[1]^ results from sustained hyperuricemia due to purine metabolism dysfunction and/or impaired uric acid excretion. Serum uric acid can form monosodium urate crystals that precipitate in synovial fluid and soft tissues, resulting in swollen and painful joints.^[2]^ The overall prevalence of gout among Chinese adults was 1.1%, with a slight increase from 1.0% to 1.3% between 2000 and 2016,^[3]^ while in Taiwan the prevalence was 6.24%.^[4]^

The first-line central strategy for effective, long-term gout management is continuous urate-lowering therapy (ULT) prescribed at a dose that achieves monosodium urate crystal dissolution, using a treat-to-serum urate target approach.^[5]^ Keeping serum urate (serum uric acid) at or below 6 mg/dL (approximately 357 μmol/L) has been widely accepted as the therapeutic target for patients with gout.^[6]^ It has also been endorsed by all internationally recognized, evidence-based gout management guidelines to date.^[7]^

Despite the fact that gout can be effectively managed using accessible and inexpensive treatments, it is not well controlled in many countries around the world, including China.^[8]^ Adherence to ULT is typically low in gout patients, ranging from 10% to 46%.^[9]^ In studies on gout treatment adherence published since 2016, Chinese gout patients have shown lower medication adherence (22%)^[8]^ compared with the United Kingdom (39%),^[10]^ Ireland (46%),^[11]^ and the United States (35.2%).^[12]^ A Turkish multicenter study reported that 68.5% of patients who received specialist education in rheumatology clinics remained adherent to ULT after a median of 7 years.^[13]^ These contrasting figures highlight the critical influence of patient education, follow-up intensity, and healthcare system characteristics on medication adherence in gout.

Adherence to medication is essential for treatment success. In 2003, the World Health Organization (WHO) proposed a multidimensional framework encompassing 5 interacting dimensions affecting adherence: socioeconomic factors, therapy-related factors, patient-related factors, condition-related factors, and health system/healthcare team–related factors.^[14]^

To our knowledge, most previous studies on this topic were cross-sectional and focused narrowly on patient-related determinants, often neglecting other elements such as therapy-related characteristics and healthcare system barriers.^[8,15]^ Furthermore, no prospective study in China has applied the WHO framework to ULT. Therefore, we conducted a 6-month and 1-year prospective cohort study to assess adherence prevalence at 6 and 12 months, examine predictors across all 5 WHO dimensions, and identify modifiable targets for early intervention. By comprehensively analyzing multilevel factors, we aim to fill this research gap and inform patient-centered strategies to improve ULT adherence in China.

## 2. Materials and methods

### 2.1. Study design

This was a 1-year observational, longitudinal, prospective study.

### 2.2. Participants

**Figure 1.**
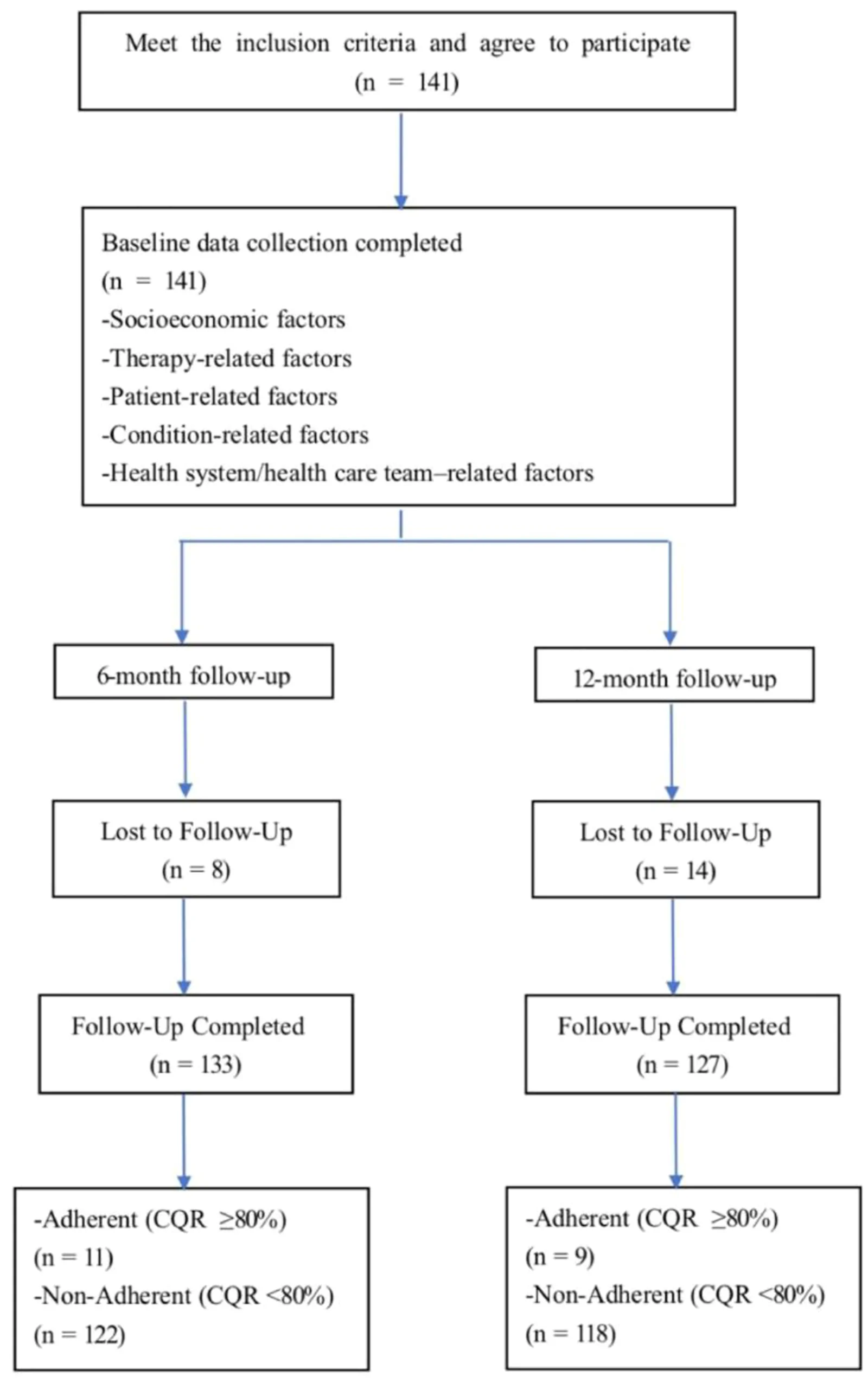
A total of 141 eligible gout patients prescribed urate-lowering therapy were enrolled at baseline. At the 6-month follow-up, 8 patients were lost to follow-up (all refused continued participation), leaving 133 patients available for analysis. Among these, 11 patients (8.3%) were classified as the adherent group (CQR score ≥ 80%) and 122 (91.7%) as the non-adherent group. At the 1-year follow-up, an additional 6 patients were lost to follow-up (all refused continued participation), resulting in 127 patients for analysis. At this time point, 9 patients (7.1%) were classified as the adherent group, and 119 (92.9%) were classified as the non-adherent group. CQR = Compliance Questionnaire in Rheumatology.

A convenience sample of participants was recruited from The First Affiliated Hospital of Chengdu Medical College from November 2019 to December 2021. The inclusion criteria were the following: meeting the gout classification criteria jointly formulated by the American College of Rheumatology and the European League Against Rheumatism in 2015^[16]^; having been prescribed at least 1 type of urate-lowering agent (ULA) at the time of enrollment, such as allopurinol, probenecid, or febuxostat; ability to speak, read, and understand Chinese; and willingness to participate in the study and provision of written informed consent. Exclusion criteria were the following: serious comorbidities such as severe infection, cardiovascular disease, respiratory disease, or kidney disease; mental illness or emotional instability precluding cooperation with the investigation; not receiving urate-lowering treatment; communication disorders; and refusal to participate in the study. A comprehensive flowchart illustrating the inclusion and exclusion criteria is presented in Figure 1.

### 2.3. Data collection and ethical considerations

Data were collected from gout patients at The First Affiliated Hospital of Chengdu Medical College. The study was approved by the Ethics Committee of The First Affiliated Hospital of Chengdu Medical College (Approval No: 2021CYFYIRB-BA-06-01).

Patients provided written consent and were assured of confidentiality and anonymity during the 30-minute data collection process.

### 2.4. Outcomes and measurements

The data collected at the first visit included socioeconomic demographic factors (gender, age, smoking and drinking history, etc), condition-related factors (course of disease, gout flare, etc), therapy-related factors (types and doses of medications used in the past week, ULT medications, etc), patient-related characteristics (anxiety, depression, treatment confidence, etc), and health system/healthcare team–related factors (physician title, visiting department). All eligible gout patients were enrolled and were followed up at 6 months and 1 year. Demographic data, including age, body mass index (BMI), smoking habits, drinking habits, and medical history, as well as data on whether new sites were involved in gout attacks, gout treatment regimens, whether medication was discontinued, and whether uric acid levels reached the target, were collected.

### 2.5. Adherence measures

During the follow-up, adherence was assessed using the Compliance Questionnaire in Rheumatology (CQR),^[17–19]^ which is the only validated one. Patients with CQR scores ≥80% were defined as adherent to ULT; those with scores <80% were defined as non-adherent.

### 2.6. Other research-related questionnaires

1. Visual Analog Scale: used for pain assessment during gout attacks. Scores range from 0 (no pain) to 10 (most unbearable pain).
2. Health Assessment Questionnaire Disability Index^[20]^: a total of 20 items; it is used to rate activity limitation in 8 domains, including dressing and grooming, arising, eating, walking, hygiene, reaching, gripping, and common daily activities, with higher scores indicating more disability.
3. Gout Knowledge Questionnaire (GKQ)^[21]^ includes 10 questions; each correct answer scores 1 point (total 10). A score ≥7 indicates good knowledge.
4. 36-Item Short-Form Questionnaire^[22]^: it contains 36 items, which measure self-reported physical and mental health across a number of domains. The 36-Item Short-Form Questionnaire includes 8 dimensions: physical functioning, pain, role limitation due to physical problems, social functioning, role limitation due to emotional problems, mental health, energy/vitality, and general health perception, all of which are used to thoroughly summarize the respondents’ quality of life. For the purpose of this study, we chose to examine only those domains related to physical health and vitality. The scoring for each domain is based on the participants’ perceived well-being. Each participant is given a score between 0 and 100 for each domain, where a score of 100 represents good health and 0 represents poor health.
5. Patient Health Questionnaire–9^[23,24]^: it is a depression screening tool that generates a total score from 0 to 27. Respondents are asked if they have been affected by 9 depression symptoms over the past 2 weeks. Scores are categorized as follows: 0 to 4 = no depression, 5 to 9 = mild depression, 10 to 14 = moderate depression, 15 to 19 = moderately severe depression, and 20 to 27 = severe depression.
6. Generalized Anxiety Disorder–7 (GAD-7)^[25,26]^: the GAD-7 is used to assess the severity of the participant’s anxiety. It is a self-report questionnaire that comprises 7 items. Respondents are asked if they have been affected by 7 anxiety symptoms over the past 2 weeks. The response to each item (e.g., “not being able to stop or control worrying”) is rated on a 4-point Likert scale. The reliability and validity of the GAD-7 have been supported by research.

### 2.7. Data analysis

Data were entered by 2 individuals. Questionnaires with >20% missing items were excluded. A double-check was used during data entry. Once all the data had been entered, 5% of them were randomly selected for accuracy verification.

After cross-checking, statistical analyses were conducted using SPSS Statistics version 25.0 (IBM Corp., Armonk). Quantitative data were expressed as mean, standard deviation, median, and interquartile range (IQR). Quantitative data were expressed as mean ± standard deviation, median, and IQR; qualitative data as frequency and percentage. Gout patients were categorized into an adherent group (CQR ≥ 80%) and a non-adherent group (CQR < 80%) according to CQR score. GKQ was entered as a binary variable (1 = poor knowledge, 2 = good knowledge). Group comparisons used chi-square tests, independent *t* tests, or Mann–Whitney *U* tests as appropriate. A multiple linear model was used to explore factors associated with CQR score. Potential confounding factors, including the concurrent use of other medications (e.g., nonsteroidal anti-inflammatory drugs, colchicine), were adjusted for in the multivariate models. Variables with a *P* value <0.10 in the bivariate analysis, along with clinically important factors such as tophi, were entered into the multiple linear regression model. Prior to the analysis, we checked the assumptions of normality, linearity, and homoscedasticity. Normality of residuals was assessed using Q-Q plots, and homoscedasticity was tested using the Durbin–Watson test. No serious violations of the assumptions were detected. The test level was 0.05. When *P* < .05, the difference was considered to be statistically significant.

Given the low prevalence of dichotomized adherence (CQR ≥ 80%) observed in our cohort, we conducted a sensitivity analysis using Firth penalized likelihood logistic regression to address potential small-sample bias associated with rare events.

## 3. Results

The study recruited 141 patients. At the 6-month follow-up, 8 patients declined to participate further, leaving 133 patients for analysis. At the 1-year follow-up, an additional 6 patients were lost, resulting in 127 patients for the final analysis. Demographic, disease-related, patient-related, treatment-related, and healthcare team–related factors are summarized separately in Tables 1 to 5.

**Table 1:** Socioeconomic demographic factors.

Characteristics	$N\;(\%)/\bar{x}\pm s$
Age	44.06 ± 15.90
Gender	
Male	136 (96.5)
Female	5 (3.5)
BMI (kg/m^2^)	26.29 ± 4.40
Work pressure	
Large	36 (25.7)
Smaller	58 (41.4)
None	46 (32.9)

Data are presented as n (%) or mean ± SD. *P* values were not applicable for this descriptive table.

BMI = body mass index, SD = standard deviation.

**Table 2:** Disease-related factors.

Characteristics	$N\;(\%)/\bar{x}\pm s$
Course of disease (yr)	6.59 ± 6.35
Family history of gout	
Not available	18 (12.8)
No	79 (56.0)
Yes	44 (31.2)
Number of joints involved in gout attacks	3.89 ± 2.90
VAS score of the latest gout attack	6.88 ± 2.35
HAQ-D1 score	4.76 ± 6.75
Hospitalized for gout in the past year	
No	106 (79.7)
Yes	27 (20.3)
Tophi	
No	73 (51.8)
Yes	68 (48.2)

Data are presented as n (%), mean ± SD.

HAQ-DI = Health Assessment Questionnaire Disability Index, IG = intercritical gout, SD = standard deviation, VAS = visual analog scale.

**Table 3:** Patient-related factors.

Characteristics	$N\;(\%)/\bar{x}\pm s$
PHQ-9	
No depression	87 (62.1)
Mild depression	39 (27.9)
Moderate depression	11 (7.9)
Moderate to severe depression	2 (1.4)
GAD-7	
Mild anxiety	27 (19.3)
No anxiety	105 (75.0)
Moderate anxiety	4 (2.9)
Moderate to severe anxiety	4 (2.9)
GKQ score	
Bad	105 (75.5)
Good	34 (24.5)

Data are presented as n (%).

GAD-7 = Generalized Anxiety Disorder–7, GKQ = Gout Knowledge Questionnaire, PHQ-9 = Patient Health Questionnaire–9.

**Table 4:** Treatment-related factors.

Characteristics	$N\;(\%)/\bar{x}\pm s$
Took NSAIDs in the past week	
No	93 (75.6)
Yes	30 (24.4)
Took Hormone in the past week	
No	117 (95.1)
Yes	6 (4.9)
Took ULA in the past week	
No	93 (75.6)
Yes	30 (24.4)
Took colchicine in the past week	
No	104 (84.6)
Yes	19 (15.4)
Initial ULT scheme	
Benzbromarone	34 (24.1)
Febuxostat	63 (44.7)
Febuxostat + benzbromarone	44 (31.2)
BMQ	
Overuse of drugs	9.27 ± 2.31
Necessity of medication	16.73 ± 3.73
Medication concerns	16.58 ± 3.46
Initial ULA dose (mg)	
20	60 (42.6)
25	33 (23.4)
40	3 (2.1)
45	43 (30.5)
50	1 (0.7)
65	1 (0.7)

Data are presented as n (%) or mean ± SD.

BMQ = Beliefs about Medicines Questionnaire, NSAIDs = nonsteroidal anti-inflammatory drugs, SD = standard deviation, ULA = urate-lowering agent, ULT = urate-lowering therapy.

**Table 5:** Medical team–related factors.

Characteristics	$N\;(\%)/\bar{x}\pm s$
Gout was first diagnosed in the Rheumatology Immunology Department	
No	112 (79.4)
Yes	29 (20.6)
The title of the first doctor in our hospital	
Primary	35 (24.8)
Advanced	82 (58.2)
Intermediate	24 (17.0)

Data are presented as n (%).

Description of socioeconomic demographic factors: the mean age of the 141 eligible participants in this study was 44.06 ± 15.90 years. Their age ranged from 16 to 86. There were 5 females (3.5%) and 136 males (96.5%). More than half (84, n = 59.5%) completed high school or above. The mean BMI was 26.29 ± 4.40 kg/m^2^.

Description of the disease-related factors: mean disease duration was 6.59 ± 6.35 years (median: 5.00, IQR: 2.00–10.00). The mean number of involved joints was 3.89 ± 2.90. Sixty-six percent experienced >2 gout flares in the previous year. Disease stages: acute gout 36.2%, chronic gout 49.6%, and intercritical gout 14.2%.

Description of the patient-related factors: eighty-one point one percent of patients with gout in this study took the following measures: taking the drug in the acute phase and stopping the drug immediately after the condition improved. Seventy-five point five percent of patients had poor GKQ scores, indicating insufficient understanding of gout. Thirty-five point eight percent of patients had some degree of depression.

Description of the treatment-related factors: in the past week, 24.4% of patients took ULA and nonsteroidal anti-inflammatory drugs, 4.9% of patients took hormonal drugs, and 15.4% took colchicine.

Description of the healthcare team–related factors: in this study, 79.4% of patients did not present to the rheumatology and immunology department at the time of the first attack of gout, but in orthopedics or other departments.

Description of the patients’ adherence in the follow-ups at 6 months and 1 year later: the patients were followed up at 6 months and 1 year later for BMI, CQR, and other information. Among patients with available data, the proportion achieving target serum urate levels was 18.0% at 6 months (n = 24/133) and 9.4% at 12 months (n = 12/127). Other results are described in Table 6.

**Table 6:** Description of adherence status of patients at the follow-up of 6 months and 1 year later.

Characteristics	6 months	1 year
	$N(\%)/median\;(IQR)/\bar{x}\pm s$	N(%)/median (IQR)
BMI	25.35 (23.69, 28.38)	25.59 (23.88, 28.41)
CQR score	56.14 (52.63, 61.40)	54.39 (45.61, 64.91)
Drug withdrawal		
No	44 (33.1)	45 (35.4)
Yes	89 (66.9)	82 (64.6)
Total ULA dose (mg/d)	23.21 ± 22.78	5.54 ± 1.77
VAS score	2.83 ± 3.14	4.80 ± 2.00
ULA		
Febuxostat/benzbromarone/allopurinol	57 (42.9)	56 (44.1)
Febuxostat + benzbromarone	25 (18.8)	9 (7.1)
No medication	49 (36.8)	62 (48.8)
Traditional Chinese medicine	2 (1.5)	0 (0)
SUA reached target level		
No	36 (27.1)	29 (22.8)
Yes	24 (18.0)	12 (9.4)
None	73 (54.9)	86 (67.7)

Data are presented as n (%), mean ± SD. Non-normally distributed data are presented as median (IQR).

BMI = body mass index, CQR = Compliance Questionnaire in Rheumatology, IQR = interquartile range, SD = standard deviation, SUA = serum uric acid, ULA = urate-lowering agent, ULT = urate-lowering therapy, VAS = visual analog scale.

### 3.1. Predictors of adherence: bivariate analysis

**Figure 2.**
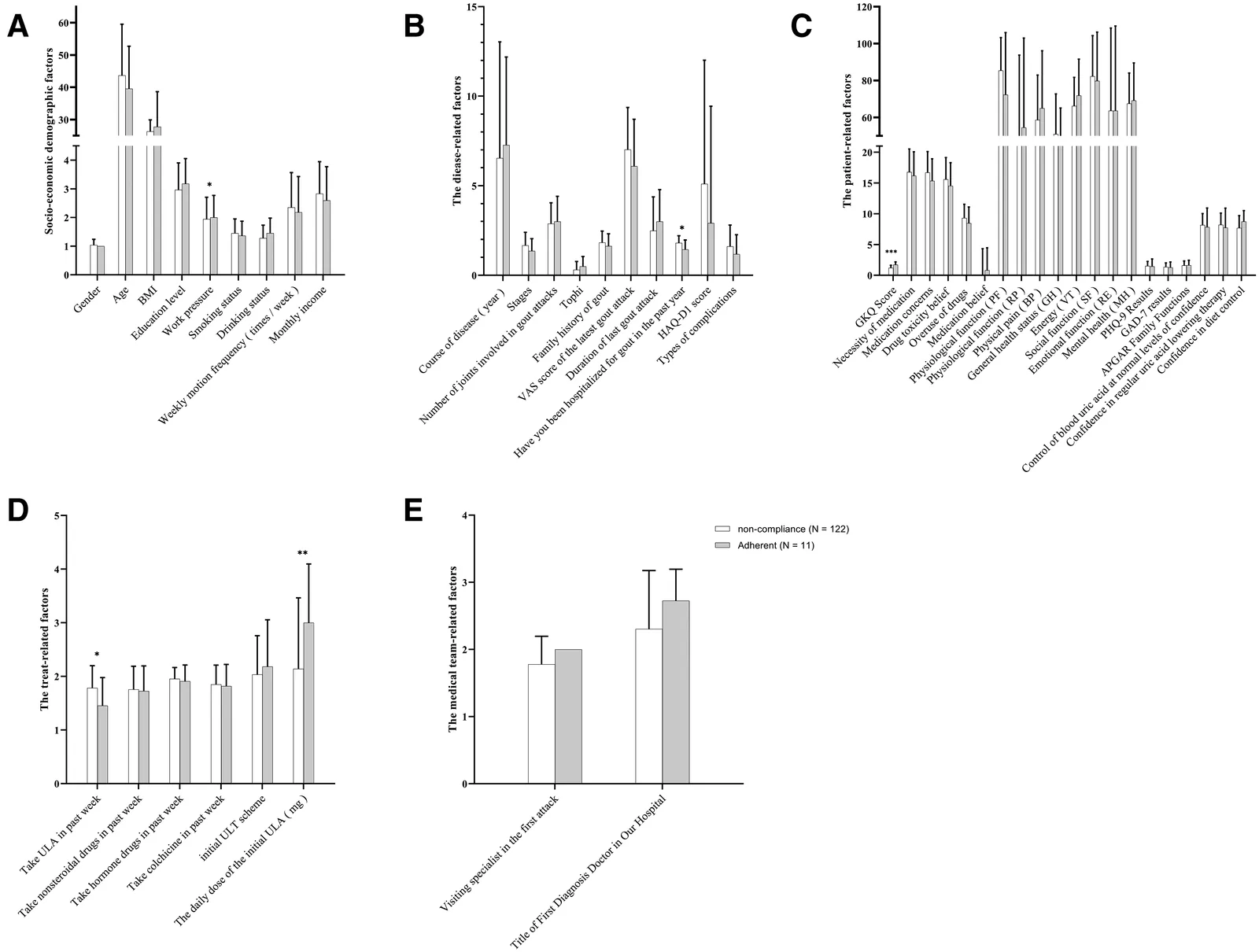
Predictors of adherence: bivariate analysis (6 months later). (A) Socioeconomic demographic factors; (B) disease-related factors; (C) patient-related factors; (D) treatment-related factors; (E) medical team–related factors. BMI = body mass index, GAD-7 = Generalized Anxiety Disorder–7, GKQ = Gout Knowledge Questionnaire, HAQ-DI = Health Assessment Questionnaire Disability Index, PHQ-9 = Patient Health Questionnaire–9, ULA = urate-lowering agent, ULT = urate-lowering therapy, VAS = visual analog scale.

In the follow-up at 6 months, the findings revealed statistically significant differences between working pressure, hospitalized for gout in the past year, GKQ score, took ULA in the past week, and the daily dose of the initial ULA (Fig. 2) and medication adherence. Larger percentages of patients with lower working pressure and higher percentages of patients with higher GKQ scores were seen in the high adherence group.

**Figure 3.**
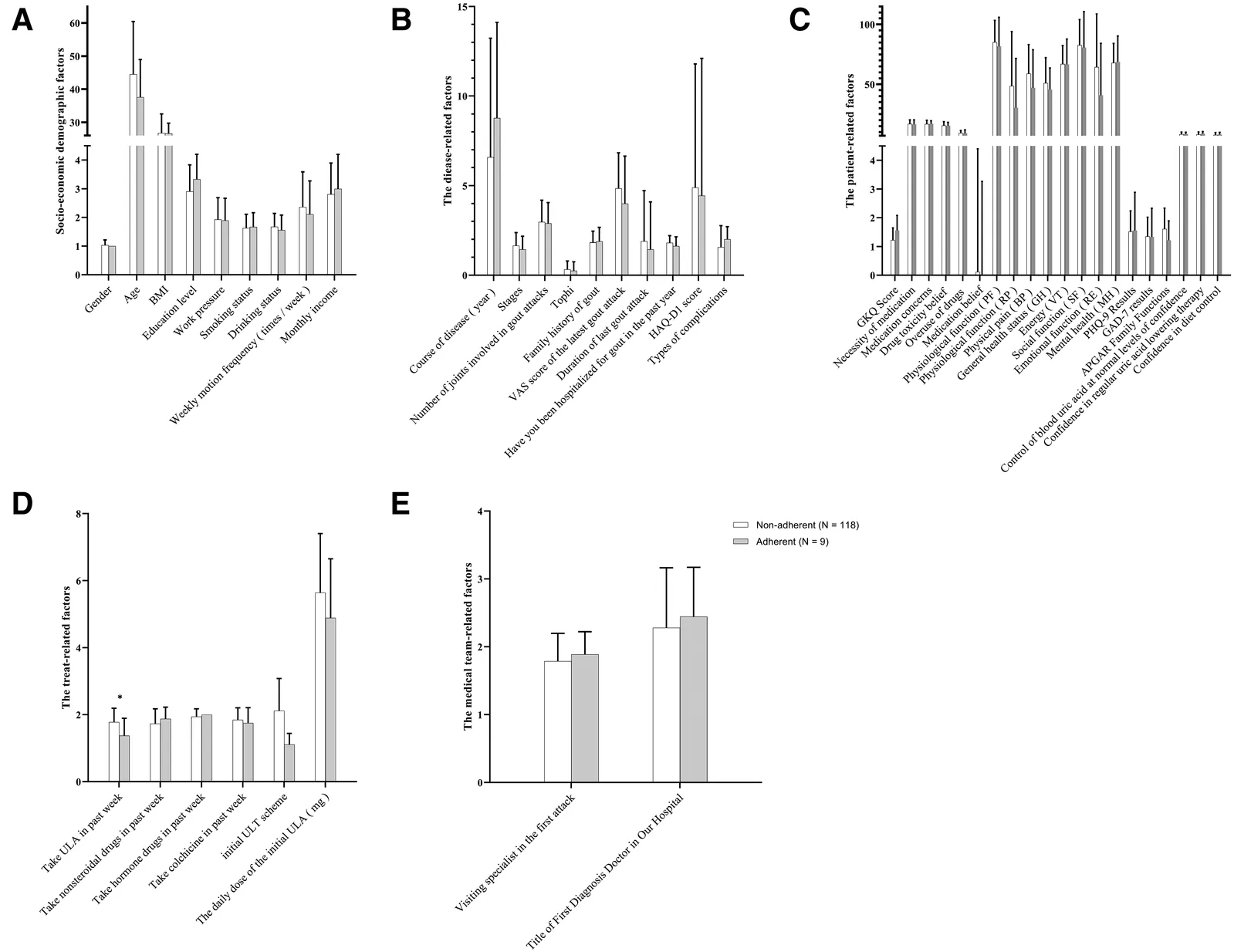
Predictors of adherence: bivariate analysis (1-year later). (A) Socioeconomic demographic factors; (B) disease-related factors; (C) patient-related factors; (D) treatment-related factors; (E) medical team–related factors. BMI = body mass index, GAD-7 = Generalized Anxiety Disorder–7, GKQ = Gout Knowledge Questionnaire, HAQ-DI = Health Assessment Questionnaire Disability Index, PHQ-9 = Patient Health Questionnaire–9, ULA = urate-lowering agent, ULT = urate-lowering therapy, VAS = visual analog scale.

In the follow-up at 1 year, there were statistically significant differences between the 2 groups in “took ULA in past week” (Fig. 3). In the past week, there were more patients taking larger doses of ULA and more patients who were taking ULA in the adherent group.

### 3.2. Predictors of adherence: multivariate analysis

In the multiple linear regression model, GKQ was treated as a categorical variable in the regression models, with the “poor knowledge” group (coded as 1) set as the reference category. GKQ score was independently associated with adherence at 6 months (β = −0.223, *P* = .001), while taking ULA in the past week was the sole predictor at 1 year (β = 0.178, *P* = .014; Tables 7 and 8).

**Table 7:** Factors related to adherence of gout patients (6 months later).

Characteristics	Unstandardized coefficient		Standardized coefficient	*P* value
	β	SE	β	
Intercept	1.903	0.209		0
Tophi	0.04	0.061	0.073	.508
ULT scheme (classification)	−0.078	0.173	−0.134	.651
ULA dose (mg)	0.007	0.059	0.035	.904
Took ULA in the past week	0.111	0.07	0.172	.116
Duration of last gout flares	−0.021	0.02	−0.11	.290
GKQ score (patients’ cognition of gout)	−0.223	0.066	−0.344	**.001**
Have you been hospitalized for gout in the past year	0.111	0.073	0.175	.13

Dependent variable: CQR score (continuous). GKQ score (binary variable: 1 = poor knowledge, 2 = good knowledge). Significant *P* values (<.05) are shown in bold.

GKQ = Gout Knowledge Questionnaire, SE = standard error, ULA = urate-lowering agent, ULT = urate-lowering therapy.

**Table 8:** Factors related to adherence of gout patients (1 year later).

Characteristics	Unstandardized coefficient		Standardized coefficient	*P* value
	β	SE	β	
Intercept	1.849	0.219		0
Tophi	0.034	0.065	0.066	.597
ULT scheme (classification)	−0.043	0.181	−0.077	.815
ULA dose (mg)	0.019	0.062	0.097	.764
Took ULA in the past week	0.178	0.071	0.298	**.014**
Last gout attack duration	−0.04	0.021	−0.22	.059
GKQ score (patients’ cognition of gout)	−0.049	0.072	−0.077	.503
Have you been hospitalized for gout in the past year	−0.041	0.078	−0.067	.596

Dependent variable: CQR score (continuous). GKQ score (binary variable: 1 = poor knowledge, 2 = good knowledge). Significant *P* value (<.05) is shown in bold.

GKQ = Gout Knowledge Questionnaire, SE = standard error, ULA = urate-lowering agent, ULT = urate-lowering therapy.

Sensitivity analysis using Firth penalized logistic regression on the binary adherence outcome confirmed the robustness of our main findings. At 6 months, a higher GKQ score remained significantly associated with adherence (odds ratio = 0.16, 95% confidence interval: 0.02–0.71, *P* = .016) (Table 9). At 1 year, taking ULA in the past week was the sole independent predictor (odds ratio = 8.24, 95% confidence interval: 1.44–63.99, *P* = .018; Table 10). These results are consistent with the multiple linear regression analysis presented above.

**Table 10:** Multivariate Firth regression analysis of factors associated with adherence at the follow-ups of 6 and 12 months.

Characteristics	6 months		1 year	
	OR (95% CI)	*P*	OR (95% CI)	*P*
Tophi	3.65 (0.61–35.13)	.163	2.76 (0.42–25.94)	.297
ULT scheme (classification)	6.28 (0.22–177.92)	.274	8.62 (0.13–426.75)	.287
ULA dose (mg)	0.48 (0.15–1.57)	.212	0.5 (0.13–2.39)	.344
Took ULA in past week	2.46 (0.43–14.14)	.304	8.24 (1.44–63.99)	**.018**
Duration of last gout flares	0.77 (0.47–1.17)	.234	0.62 (0.34–1.00)	.051
GKQ score (patients’ cognition of gout)	0.16 (0.02–0.71)	**.016**	0.45 (0.07–2.69)	.371
Have you been hospitalized for gout in the past year	1.63 (0.26–9.08)	.583	0.58 (0.08–3.78)	.574

GKQ score (binary variable: 1 = poor knowledge, 2 = good knowledge). Significant *P* values (<.05) are shown in bold.

CI = confidence interval, GKQ = Gout Knowledge Questionnaire, OR = odds ratio, ULA = urate-lowering agent, ULT = urate-lowering therapy.

**Table 9:** Univariate Firth regression analysis of factors associated with adherence at the follow-up of 6 months and 1 year later.

Characteristics	6 months		1 year	
	OR (95% CI)	*P*	OR (95% CI)	*P*
Gender	1.08 (0.11–144.67)	.961	0.75 (0.07–101.74)	.854
Age	1.02 (0.98–1.07)	.275	1.03 (0.98–1.09)	.219
BMI	0.99 (0.88–1.17)	.848	0.93 (0.84–1.06)	.21
Education level	2.71 (0.02–44.00)	.58	2.71 (0.02–44.00)	.58
Work pressure	0.18 (0.02–0.82)	**.025**	0.95 (0.20–4.16)	.951
Smoking status	0.94 (0.28–3.23)	.919	4.95 (1.06–47.56)	**.040**
Drinking status	1.02 (0.30–4.35)	.971	1.33 (0.33–7.37)	.703
Weekly motion frequency (times/wk)	1.13 (0.17–12.15)	.904	1.41 (0.24–14.76)	.718
Monthly income	1.09 (0.08–14.24)	.941	0.76 (0.07–6.24)	.8
Course of disease (yr)	0.98 (0.90–1.08)	.588	0.95 (0.88–1.05)	.282
Stages	2.70 (0.70–14.81)	.154	1.93 (0.47–10.92)	.375
Number of joints involved in gout attacks	0.91 (0.76–1.11)	.309	0.89 (0.74–1.11)	.286
Tophi	2.86 (0.84–12.04)	.096	2.05 (0.55–8.97)	.284
Family history of gout	1.85 (0.52–6.65)	.335	1.44 (0.31–6.23)	.627
VAS score of the latest gout attack	1.17 (0.91–1.49)	.215	1.01 (0.75–1.33)	.949
Duration of last gout attack	1.78 (0.13–253.36)	.702	1.84 (0.13–262.33)	.684
Have you been hospitalized for gout in the past year	5.13 (1.35–20.58)	**.017**	2.71 (0.59–11.03)	.187
HAQ-DI score	1.04 (0.95–1.21)	.409	1.00 (0.92–1.12)	.937
GKQ score	0.12 (0.03–0.40)	**.001**	0.24 (0.06–0.90)	**.035**
Necessity of medication	1.05 (0.89–1.20)	.508	1.04 (0.85–1.23)	.663
Medication concerns	1.10 (0.95–1.26)	.186	1.00 (0.78–1.20)	.985
Drug toxicity belief	1.08 (0.92–1.25)	.316	1.02 (0.82–1.24)	.826
Overuse of drugs	1.16 (0.90–1.47)	.233	1.06 (0.77–1.44)	.697
Medication belief	0.96 (0.83–1.11)	.555	1.03 (0.88–1.22)	.739
Physiological function (PF)	1.03 (1.00–1.05)	**.044**	1.01 (0.98–1.04)	.48
Physiological function (RP)	1.00 (0.98–1.01)	.639	1.01 (0.99–1.03)	.278
Physical pain (BP)	0.99 (0.96–1.01)	.432	1.02 (0.99–1.05)	.176
General health status (GH)	1.00 (0.98–1.04)	.838	1.01 (0.98–1.05)	.576
Energy (VT)	0.98 (0.93–1.02)	.296	1.00 (0.96–1.04)	.868
Social function (SF)	1.01 (0.98–1.03)	.635	1.01 (0.97–1.03)	.676
Emotional function (RE)	1.00 (0.99–1.01)	.942	1.01 (1.00–1.03)	.131
Mental health (MH)	1.00 (0.96–1.03)	.858	1.00 (0.96–1.04)	.983
PHQ-9 results	3.19 (0.69–30.54)	.149	2.48 (0.51–24.19)	.281
GAD-7 results	6.29 (0.76–818.54)	.1	4.76 (0.56–623.45)	.186
APGAR family functions	1.12 (0.30–4.93)	.87	10.31 (1.22–1346.52)	**.028**
Control of blood uric acid at normal levels of course	9.00 (0.15–3674.77)	.306	1.67 (0.01–406.24)	.821
Confidence in regular uric acid-lowering therapy	21.00 (0.46–7932.16)	.123	2.33 (0.01–554.50)	.705
Confidence in diet control	1.40 (0.01–307.03)	.877	1.00 (0.00–224.94)	1
Take ULA in the past week	4.25 (1.24–15.10)	**.022**	5.45 (1.35–25.07)	**.018**
Take nonsteroidal drugs in the past week	1.27 (0.29–4.46)	.729	0.54 (0.05–2.62)	.474
Take hormone drugs in the past week	2.66 (0.26–15.27)	.355	0.89 (0.01–8.74)	.938
Take colchicine in the past week	1.46 (0.26–5.78)	.629	2.06 (0.35–8.96)	.382
Initial ULT scheme	1.92 (0.39–9.57)	.412	0.81 (0.08–5.18)	.828
The daily dose of the initial ULA (mg)	0.22 (0.02–1.41)	.11	0.87 (0.11–9.86)	.897
Visiting specialist in the first attack	0.91 (0.17–3.47)	.903	0.65 (0.07–3.08)	.619
Title of first diagnosis doctor in our hospital	0.08 (0.00–0.92)	**.042**	0.22 (0.02–1.44)	.115

GKQ score (binary variable: 1 = poor knowledge, 2 = good knowledge). Significant *P* values (<.05) are shown in bold.

BMI = body mass index, CI = confidence interval, GAD-7 = Generalized Anxiety Disorder-7, GKQ = Gout Knowledge Questionnaire, HAQ-DI = Health Assessment Questionnaire Disability Index, OR = odds ratio, PHQ-9 = Patient Health Questionnaire-9, ULA = urate-lowering agent, ULT = urate-lowering therapy, VAS = visual analog scale.

## 4. Discussion

This is the first study in China to explore the factors influencing adherence to ULT in gout patients from the perspective of the 5 factors influencing adherence proposed by the WHO in 2003. Our study showed the region’s adherence was lower than the global adherence figures for gout patients (10%–46%). At the 6-month follow-up, only 8.3% of patients adhered to ULT, but 1 year later, this figure had fallen to 7.1%. This downward trend is consistent with an Italian general medicine study^[27]^ that showed a decreasing trend in medication adherence in gout patients as treatment progressed.

However, adherence rates vary substantially across different healthcare settings. A Spanish prospective cohort reported an adherence rate of 82.1% among patients who received structured education and empowerment interventions.^[28]^ Similarly, a Norwegian prospective study (NOR-Gout) implemented a treat-to-target strategy with tight control and monthly visits during the first year; at 5 years, patients reported high adherence with a median Medication Adherence Rating Scale-5 score of 24 (out of 25), although 26.6% were classified as non-adherent (Medication Adherence Rating Scale-5 ≤ 21)^.[29]^ The wide disparity between our findings (7%–8% at 6 and 12 months) and these European cohorts likely reflects differences in patient education, healthcare system supports (e.g., universal coverage, electronic prescription monitoring), and adherence measurement methods (self-report vs objective pharmacy data). Nonetheless, our findings represent the real-world adherence status among Chinese gout patients, where systematic patient education and regular specialist follow-up are not routinely provided.

In the univariate analysis examining factors associated with ULT adherence among gout patients, the following variables showed significant correlations at the 6-month follow‑up: work pressure, taking ULA in the past week, GKQ score, hospitalization for gout in the previous year, and ULA dose. At the 1-year follow-up, only taking ULA in the past week was significantly correlated with medication adherence to ULT. After considering the effect of the above single factors on medication adherence in this study, further analysis in a multiple linear regression model revealed that the following factors remained associated with medication adherence in patients with gout: GKQ score (6 months later) and taking ULA in the past week (1 year later).

GKQ score was an independent predictor of adherence at 6 months. Patients with better gout knowledge were more likely to adhere to ULT.^[15]^ If the patient is unaware of the effects of allopurinol and the cause of the gout attack, it will result in low medication adherence.^[2]^ In our study, only 24.5% of patients had a good perception about gout, knowing that the disease required long-term treatment and management, which promoted medication adherence in patients with gout. However, this association diminished at 1 year, possibly due to reduced gout flare frequency and decreased attention to disease management.

Patients who took ULA in the past week showed higher medication adherence. Gout, as a chronic inflammatory arthritis, is commonly treated clinically to lower uric acid, mainly including 2 types of drugs: inhibiting uric acid production and promoting uric acid excretion. Chinese gout patients can learn about it through various web pages and apps, and buy drugs in pharmacies or online. This promotes patients’ understanding and acceptance of ULT, and affects the patients’ medication adherence to a certain extent. Persistent untreated gout can result in serious adverse outcomes, including recurrent gouty arthritis, chronic gouty arthropathy, tophi, and urolithiasis. Healthcare professionals should seek to not only understand patients’ underlying beliefs about their gout and preconceived ideas of ULT, but also explain the necessity and benefits of ULT, besides addressing patient concerns on potential side effects of prescribed prophylactic therapy for gout flares.

A notable proportion of patients discontinued ULT: 66.9% within 6 months and 64.6% at 1 year. Common reasons included absence of recurrence (30.1%–46.3%), side effects, coronavirus disease 2019 (COVID‑19) pandemic impacts, high drug costs, and inconvenience of follow-up.

In 2020, COVID-19 spread rapidly around the world. On January 30, 2020, the WHO announced that COVID-19 was listed as an international public health emergency, and on March 11, it was declared a pandemic.^[30]^ During this period, there were problems such as interruption or delay of appointments during medical treatment.^[31]^ A survey^[32]^ shows that 48% of patients cancel or postpone medical appointments, 24% call for medical treatment, 14% change the type or dose of drugs themselves, 10% cannot get drugs, 4% cannot even reach the hospital for medical treatment, and so on. This is the reason for drug withdrawal that has not appeared in previous studies.

This study has several limitations. First, the single‑center design and modest sample size may limit the generalizability of the findings. Second, self‑reported adherence measures are susceptible to recall bias, which could affect data accuracy. Future multicenter studies with larger samples and objective adherence assessments, such as pill counts or pharmacy records, are recommended. Third, by 1-year follow-up, there were only 127 cases. As this study was a single-center study, the persuasiveness of the results needs to be further strengthened. Fourth, in this study, patients’ self-report was used to collect some data of gout patients, which might have recall bias or social desirability bias; although we repeatedly emphasized the purpose of the study to encourage active cooperation, this limitation remains. Fifth, the 1-year follow-up, while prospective, may be insufficient to capture long-term adherence patterns and the factors influencing them over a longer horizon. Moreover, the study period coincided with the peak of the COVID-19 pandemic, which caused significant disruptions in healthcare access and medication continuity, as acknowledged in our results. This extraordinary context may limit the generalizability of our findings to non-pandemic settings, and future research should examine adherence patterns in post-pandemic periods.

## 5. Conclusion

Medication adherence to ULT among gout patients in this Chinese cohort is notably low and declines over time. Patient knowledge and recent medication-taking behavior are key modifiable predictors. These findings underscore the importance of cognitive and behavioral factors in adherence models for chronic diseases and highlight that early, targeted educational and behavioral interventions within the first 6 months of treatment are urgently needed to improve adherence in Chinese gout patients.

## Acknowledgments

We would like to thank Xuan Lei, Ting Yi, and Yapeng Lin for their contributions to study design, data acquisition, and analysis. We also thank all investigators and site staff of The First Affiliated Hospital of Chengdu Medical College who participated in this study. No writing assistance was received in the preparation of this manuscript. All named individuals have provided their written consent to be acknowledged.

## Author contributions

**Conceptualization:** Haiyan Jiang, Yanda Yang, Xuan Lei, Ting Yi, Xiran Luo, Shuya Xiong, Rong Wang, Yapeng Lin.

**Data curation:** Haiyan Jiang, Yanda Yang, Xuan Lei, Ting Yi, Weiyu Yang, Shuya Xiong, Rong Wang, Zhaoxia Ran, Yapeng Lin.

**Methodology:** Haiyan Jiang, Yanda Yang, Ting Yi, Xiran Luo, Weiyu Yang, Rong Wang, Yapeng Lin.

**Visualization:** Haiyan Jiang, Yanda Yang, Xuan Lei, Xiran Luo, Weiyu Yang, Shuya Xiong, Rong Wang, Tao Lu.

**Investigation:** Xuan Lei, Yuanjun Xie, Liwen Yao.

**Resources:** Xuan Lei, Zepeng Lai.

**Supervision:** Ting Yi, Tao Lu, Yapeng Lin.

**Software:** Yuanjun Xie, Zepeng Lai.

**Formal analysis:** Liwen Yao.

**Project administration:** Zhaoxia Ran.

**Writing – original draft:** Haiyan Jiang, Yanda Yang, Yapeng Lin.

**Writing – review & editing:** Haiyan Jiang, Yanda Yang, Xuan Lei, Ting Yi, Xiran Luo, Weiyu Yang, Shuya Xiong, Rong Wang.

## References

[1] Dehlin M Jacobsson L Roddy E. Global epidemiology of gout: prevalence, incidence, treatment patterns and risk factors. Nat Rev Rheumatol. 2020;16:380–90. 10.1038/s41584-020-0441-1 32541923

[2] Spencer K Carr A Doherty M. Patient and provider barriers to effective management of gout in general practice: a qualitative study. Ann Rheum Dis. 2012;71:1490–5. 10.1136/annrheumdis-2011-200801 22440822

[3] Chen Y Tang Z Huang Z. The prevalence of gout in mainland China from 2000 to 2016: a systematic review and meta-analysis. J Public Health. 2017;25:521–9.

[4] Kuo C Grainge MJ See L. Epidemiology and management of gout in Taiwan: a nationwide population study. Arthritis Res Ther. 2015;17:13. 10.1186/s13075-015-0522-8 PMC4342824 25612613

[5] Dalbeth N Gosling AL Gaffo A Abhishek A. Gout. Lancet. 2021;397:1843–55. 10.1016/S0140-6736(21)00569-9 33798500

[6] Nazia R Brian W C Yi-Lin W. Modifiable factors associated with allopurinol adherence and outcomes among patients with gout in an integrated healthcare system. J Rheumatol. 2015;42:504–12. 10.3899/jrheum.140588 PMC4922495 25512479

[7] FitzGerald John D Nicola D Ted M. 2020 American College of Rheumatology guideline for the management of Gout. Arthritis Rheumatol. 2020;72:879–95. 10.1002/art.41247 32390306

[8] Sheng F Fang W Zhang B Sha Y Zeng X. Adherence to gout management recommendations of Chinese patients. Medicine (Baltimore). 2017;96:e8532. 10.1097/MD.0000000000008532 PMC5690752 29137059

[9] De Vera MA Marcotte G Rai S Galo JS Bhole V. Medication adherence in gout: a systematic review. Arthritis Care Res (Hoboken). 2014;66:1551–9. 10.1002/acr.22336 24692321

[10] Scheepers L Burden AM Arts ICW. Medication adherence among gout patients initiated allopurinol: a retrospective cohort study in the Clinical Practice Research Datalink (CPRD). Rheumatology (Oxford). 2018;57:1641–50. 10.1093/rheumatology/key155 29893941

[11] McGowan B Bennett K Silke C Whelan B. Adherence and persistence to urate-lowering therapies in the Irish setting. Clin Rheumatol. 2016;35:715–21. 10.1007/s10067-014-2823-8 25409858

[12] Riggs KR Richman JS Cherrington AL Singh JA. Allopurinol adherence in US patients with Gout: analysis of the Medical Expenditure Panel Survey, 2018-2021. J Clin Rheumatol. 2025;31:83–6. 10.1097/RHU.0000000000002177 PMC12160576 39607067

[13] Görgülü MB Kardaş RC Şeramet DK. Evaluation of treatment compliance in gout patients: a patient-centered study. Turk J Med Sci. 2025;55:413–22. 10.55730/1300-0144.5985 PMC12058013 40342319

[14] De Geest S Sabaté E. Adherence to long-term therapies: evidence for action. Eur J Cardiovasc Nurs. 2003;2:323. 10.1016/S1474-5151(03)00091-4 14667488

[15] Yin R Cao H Fu T. The rate of adherence to urate-lowering therapy and associated factors in Chinese gout patients: a cross-sectional study. Rheumatol Int. 2017;37:1187–94. 10.1007/s00296-017-3746-x 28551724

[16] Neogi T Jansen TL Dalbeth N. 2015 Gout classification criteria: an American College of Rheumatology/European League Against Rheumatism collaborative initiative. Arthritis Rheumatol. 2015;67:2557–68. 10.1002/art.39254 PMC4566153 26352873

[17] de Klerk E van der Heijde D Landewé R van der Tempel H van der Linden S. The compliance-questionnaire-rheumatology compared with electronic medication event monitoring: a validation study. J Rheumatol. 2003;30:2469–75. 14677194

[18] Klerk ED Heijde D Tempel H Linden S. Development of a questionnaire to investigate patient compliance with antirheumatic drug therapy. J Rheumatol. 1999;26:2635. 10606375

[19] Shi PL Wu ZZ Wu L Gao RC Wu ZG Wu GC. Reliability and validity of the Chinese version of the eight-item Morisky Medication Adherence Scale in Chinese patients with systemic lupus erythematosus. Clin rheumatol. 2022;41:2713–20. 10.1007/s10067-022-06195-y 35536414

[20] Dalbeth N House ME Horne A Petrie KJ McQueen FM Taylor WJ. Prescription and dosing of urate-lowering therapy, rather than patient behaviours, are the key modifiable factors associated with targeting serum urate in gout. BMC Musculoskelet Disord. 2012;13:174. 10.1186/1471-2474-13-174 PMC3493372 22978848

[21] Zhang LY Schumacher HR Su HH. Development and evaluation of a survey of gout patients concerning their knowledge about gout. J Clin Rheumatol. 2011;17:242–8. 10.1097/RHU.0b013e318228b4e2 21778899

[22] Ware JE Jr Sherbourne CD. The MOS 36-item short-form health survey (SF-36). I. Conceptual framework and item selection. Med Care. 1992;30:473–83. 1593914

[23] Suni JH Virkkunen T Husu P Tokola K Parkkari J Kankaanpää M. Reliability and construct validity of the modified Finnish version of the 9-item patient health questionnaire and its associations within the biopsychosocial framework among female health-care workers with sub-acute or recurrent low back pain. BMC Musculoskelet Disord. 2021;22:37. 10.1186/s12891-020-03832-y PMC7792227 33413235

[24] Kroenke K Spitzer RL Williams JB. The PHQ-9: validity of a brief depression severity measure. J Gen Intern Med. 2001;16:606–13. 10.1046/j.1525-1497.2001.016009606.x PMC1495268 11556941

[25] Shen Y Yuan S Liu J. The reliability, validity and screening effect of the happiness index scale among inpatients in a general hospital. BMC Psychiatry. 2022;22:601. 10.1186/s12888-022-04219-0 PMC9463772 36085028

[26] Spitzer RL Kroenke K Williams JB Löwe BJ. A brief measure for assessing generalized anxiety disorder: the GAD-7. Arch Internal Med Arch Intern Med. 2006;166:1092–7. 10.1001/archinte.166.10.1092 16717171

[27] Mantarro S Capogrosso-Sansone A Tuccori M. Allopurinol adherence among patients with gout: an Italian general practice database study. Int J Clin Pract. 2015;69:757–65. 10.1111/ijcp.12604 25683693

[28] Perez-Ruiz F Perez-Herrero N Richette P Stack AG. High rate of adherence to urate-lowering treatment in patients with gout: who’s to blame? Rheumatol Ther. 2020;7:1011–9. 10.1007/s40744-020-00249-w PMC7695758 33111171

[29] Uhlig T Karoliussen LF Sexton J. Non-adherence to urate lowering therapy in gout after 5 years is related to poor outcomes: results from the NOR-Gout study. Rheumatology (Oxford). 2025;64:1799–806. 10.1093/rheumatology/keae514 PMC11962959 39292608

[30] Favalli EG Ingegnoli F De Lucia O Cincinelli G Cimaz R Caporali R. COVID-19 infection and rheumatoid arthritis: faraway, so close! Autoimmun Rev. 2020;19:102523. 10.1016/j.autrev.2020.102523 PMC7102591 32205186

[31] Sirotich E Dillingham S Grainger R Hausmann JS. Capturing patient-reported outcomes during the COVID-19 pandemic: development of the COVID-19 global rheumatology alliance patient experience survey. Arthritis Care Res (Hoboken). 2020;72:871–3. 10.1002/acr.24257 PMC7272930 32386125

[32] Kaleb M Kristin W Yomei S. Experiences of patients with rheumatic diseases in the United States during early days of the COVID-19 pandemic. ACR Open Rheumatol. 2020;2:335–43. 10.1002/acr2.11148 PMC7264613 32311836

